# Pretreatment HIV‐1 Drug Resistance Among Newly Diagnosed People in Eastern Ethiopia

**DOI:** 10.1002/hsr2.70672

**Published:** 2025-04-16

**Authors:** Abdella Gemechu, Adane Mihret, Mesfin Mengesha, Dawit Hailu Alemayehu, Eleni Kidane, Abraham Aseffa, Rawleigh Howe, Berhanu Seyoum, Andargachew Mulu

**Affiliations:** ^1^ School of Medical Laboratory Sciences, College of Health and Medical Sciences Haramaya University Harar Ethiopia; ^2^ Armauer Hansen Research Institute Addis Ababa Ethiopia; ^3^ Ethiopian Public Health Institute Addis Ababa Ethiopia

**Keywords:** Ethiopia, HIV‐1, pretreatment drug resistance

## Abstract

**Background and Aims:**

In Ethiopia, HIV‐1 pretreatment drug resistance (PDR) data are limited owing to a lack of routine genotyping resistance tests. This study aimed to determine the prevalence of HIV‐1 PDR mutations and genetic diversity among newly diagnosed people with HIV in eastern Ethiopia.

**Methods:**

HIV RNA was extracted using Abbott m2000sp. HIV‐1 partial pol genes were amplified and sequenced using the Sanger dideoxy method. DRM profiles were examined and interpreted according to the calibrated population resistance (CPR) and Stanford University HIV drug resistance algorithms. A maximum likelihood phylogenetic tree was constructed using PhyML version 3.0 and visualized using the iTOL tool. Bivariable and multivariable logistic regression models were used to identify baseline factors associated with outcomes at a *p* value of < 0.05.

**Results:**

Among newly diagnosed individuals with baseline viral load (≥ 1000 copies/mL) and amplifications were successful, the genotyping success rate was 78.4%. Among the isolates successfully sequenced, three HIV‐1 strains were detected, of which 97.1% had HIV‐1 subtype C, 1.4% A1C, and 1.4% CF1 recombinant. According to the Stanford HIVDR algorithm, 21.7% of people had at least one drug associated PDR mutation, whereas CPR reported 14.5% DRMs. NNRTIs had the highest PDR mutation rate (13.0%), followed by NRTIs (7.2%) and PIs (2.9%). The most commonly observed major DRMs were: NNRTIs (K103N and G190A), NRTIs (D67G and L210W), and PIs (L90M and I54S). Patients who were bedridden at enrollment were more likely to harbor PDR mutations (AOR: 5.4; 95% CI: 1.53–30.7).

**Conclusions:**

High PDR levels, predominantly for NNRTIs, are observed. During clinical follow‐up, special attention should be given to bedridden functional status patients. Further surveillance studies are needed to evaluate the long‐term effects of prolonged accumulation of resistance and its transmission on current ART regimens and to design appropriate interventions to halt the HIV epidemic.

## 1 Introduction

The worldwide expansion of antiretroviral treatment (ART) has significantly reduced the morbidity and mortality associated with HIV [1]. However, the emergence of HIV‐1 drug resistance (HIVDR) is a major bottleneck in long‐term HIV treatment and prevention [2]. HIVDR has significant clinical and public health implications for increasing the risk of first‐line treatment failure and onward transmission of drug‐resistant strains to treatment‐naive individuals [3]. Pretreatment drug resistance (PDR) can increase the chance of virological failure and compromise the long‐term effectiveness of recommended ART regimens [4, 5]. This is a real challenge in ending the HIV epidemic, particularly in resource‐limited settings where the prevalence of HIV‐1 is high, antiretroviral (ARV) drugs with low genetic barriers to resistance are commonly used, and no routine drug resistance testing is available [3, 6].

The World Health Organization (WHO) recommends periodic HIVDR surveys to inform treatment strategies [7]. According to the latest WHO HIVDR Report, the prevalence of acquired and transmitted HIVDR in ART‐naive individuals has increased exponentially in recent years, becoming an important obstacle to ending the HIV‐1 epidemic as a public health threat by 2030 [8]. The report indicated that 10% of adults starting HIV treatment were resistant to nonnucleoside reverse transcriptase inhibitors (NNRTIs), whereas people with previous exposure to ARV drugs were three times more likely to present resistance to the NNRTI drug class. Similarly, a recent systematic review and meta‐analysis conducted in eastern Africa reported a pooled PDR of 10.0%, of which 9.4%, 2.6%, and 0.7% were attributable to the use of NNRTIs, NRTIs, and PIs, respectively [9].

Because of the high mutation rate and replicative capacity of HIV, as well as its ability to establish lifelong infections and large‐scale pandemics, viral strains have diversified significantly over time [10, 11]. HIV‐1 genetic diversity may influence global HIV clade distribution, disease progression, vaccine design, pathogenesis, diagnostic test performance, and viral load measures. Moreover, it affects ART effectiveness, drug resistance mutation (DRM) patterns, and the emergence of drug‐resistant strains [12]. To date, HIV‐1 M (the “pandemic”) has been classified into ten subtypes (A–D, F–H, and J–L), six A (A1–A6), and two F (F1–F2) sub‐subtypes, 132 CRFs, and many URFs [13, 14, 15, 16].

Sub‐Saharan Africa has a diverse range of HIV‐1 M subtypes and CRFs, with Central and West African countries having the greatest genetic diversity of HIV‐1 [13, 17]. Globally, subtype C is the most common and prominent subtype in Eastern Africa, Southern Africa, and India [13]. Moreover, the information obtained from the genetic diversity of HIV‐1 can improve our understanding of HIV‐1 infections and their evolutionary and epidemiological aspects in the region. This, in turn, may inform the design of epidemic control measures tailored to HIV‐1 transmission [18]. Therefore, it is critical to track genetic diversity and DRMs, monitor changes in the epidemiology of the virus and design appropriate measures for its control.

In Ethiopia, there have been few studies on PDR in various parts of the country, with the overall prevalence of PDR among different populations ranging from 2.2% to 16.5% [19, 20, 21, 22, 23, 24]. According to a recent meta‐analysis, the pooled prevalence of PDR in Ethiopia is 4.6%–4.8% [9, 25]. However, in the current study area, there was a lack of data on PDR and DRMs among newly diagnosed people with HIV. In this study, we sought to determine the prevalence of HIV‐1 PDR mutations and genetic diversity among newly diagnosed people with HIV in eastern Ethiopia.

## 2 Methods

### 2.1 Study Setting and Population

This prospective cohort follow‐up study was conducted at 15 health facilities in eastern Ethiopia. The study population consisted of all newly diagnosed HIV‐positive people who began first‐line ART. The health facilities were from Harari and Somali Regional State, Dire Dawa City, and the East and West Hararghe zones of the Oromia region. The study sites: Chiro (319 km), Deder (438 km), Dire Dawa (514 km), Harar (526 km), Bisidimo (551 km), and Jigjiga (628 km) were far away east of Addis Ababa, the capital of Ethiopia. The sample size was calculated using the standard WHO HIV PDR sample size calculator [26] with the following assumptions: an estimate of prevalence of HIVDR among all ART initiators of 10%, a genotyping failure rate of 20%, a precision of the HIVDR prevalence estimate of 5%, and 15 ART clinic models. Study participants were consecutive enrolled between October 2020 and December 2021.

### 2.2 Data Collection

A structured questionnaire and checklists were designed to collect sociodemographic factors, clinical data, laboratory parameters, and information regarding the initiated first‐line ART regimen. Face‐to‐face interviews were conducted with study participants or caregivers/guardians of children/adolescents under the age of 18 years.

### 2.3 Sample Collection and Processing

At the start of the ART, 5 mL of whole blood was collected from each participant in vacutainer tubes containing EDTA. Plasma samples were harvested by centrifugation of whole blood at 2050 *g* for 5 min, aliquoted into cryotubes, and transported at 4°C in a cold chain system to Harari Health Research and Regional Laboratory, Harar, Ethiopia, for HIV‐1 RNA VL testing. The plasma samples were stored in a deep freezer (−80°C) until testing at Haramaya University, College of Health and Medical Sciences, CHAMPS Microbiology Laboratory.

### 2.4 Viral Load Determination and RNA Extraction

To extract HIV‐1 RNA and determine plasma VL, the Abbott m2000sp automated sample preparation system and the Abbott m2000rt Quantitative Abbott Real‐Time HIV‐1 assay (Abbott Molecular Inc., Des Plaines, IL, USA) were used. The thawed plasma sample volume (200 µL) was used as the initial input for VL quantification and HIV RNA extraction for genotypic HIVDR testing.

### 2.5 Complementary DNA (cDNA) Synthesis, RT‒PCR, and Nested PCR

Viral cDNA synthesis was carried out using 8 μL of the RNA eluted in a final reaction volume of 20 μL. This template was first annealed with a HIVRevINT‐specific primer in a reaction with a 25 mM deoxyribonucleotide triphosphate (dNTP) mixture and DEPC‐treated water. This mixture was then combined with another reverse transcription reaction tube containing a master mix of 5× Superscript IV (SSIV) buffer, 100 mM dithiothreitol (DTT), RNase/RiboLock, and the SSIV reverse transcriptase (RT) enzyme (Invitrogen, Life Technologies, USA) under the thermal conditions listed in Supporting Information: Table S1. This reaction mixture was immediately used for polymerase chain reaction (PCR) amplification or stored at −20°C. The assay protocol was adopted from previous studies [23].

A portion of the HIV‐1 pol gene [including the entire HIV protease (PR:1–99) and a portion of reverse RT spanning a minimum of codons 1–327] was amplified via two‐step PCR using Platinum Taq High Fidelity DNA polymerase enzyme (Invitrogen, Life Technologies, USA). In each PCR step, two sets of outer and inner in‐house primers were used for first‐round PCR and nested PCR, respectively (Supporting Information: Table S1). The reaction mixture volume was 50 µL for both PCR steps, and the cycling conditions were the same as those indicated in the table. The expected amplicon size from the first‐round PCR was 1758 base pairs (bp) [23]. Thereafter, nested PCR was performed using 3 µL of the first‐round PCR DNA product as a template with the primer set forward (HIVSeqA primer: AGC CAA CAG CCC CAC CAG) and reverse (HIVSeqH primer: CTG TAT TTC TGC TAT TAA GTC TTT TG) to make a 50 µL volume reaction using the same cycling conditions as in Supporting Information: Table S1. The expected amplicon size of the nested PCR product was 1390 bp [23], as confirmed by 1.5% agarose gel electrophoresis with ethidium bromide staining, and visualized under ultraviolet light. The quality and concentration of the purified DNA were checked using gel electrophoresis and Nanodrop, respectively.

### 2.6 Purification of PCR Products

The final nested PCR product was purified using the PureLink Quick Gel Extraction and PCR Purification Combo Kit according to the manufacturer's instructions. The final volume of eluted purified DNA was 50 µL and stored at −20°C for long‐term storage.

### 2.7 DNA Sequencing and Purification

The purified nested PCR amplicons were sequenced by Sanger DNA Sequencing using the BigDye Terminator v3.1 Cycle Sequencing Kit (Applied Biosystems). Four specific primers used for nested PCR (inner in‐house primers) (1 µM each). These primers were: HIVpcrFor2: 5′‐AGC CAA CAG CCC CAC CAG‐3′ [HXB2 location 2150–2167]; HIVSeq. 1: 5′‐GTT AAA CAA TGG CCA TTG ACAGA‐3′ [HXB2 location 2610–2632]; HIVSeq. 4: 5′‐CCA TCC CTG TGG AAG CAC ATT‐3′ [HXB2 location 2988–3008]; and HIVpcrRev2: 5′‐CTG TAT TTC TGC TAT TAA GTC TTT TG‐3′ [HXB2 location, 3514–3539]. The total volume of the cycle sequencing reaction was 20 µL, containing 3 µL of purified DNA, 3.2 µL of 1 µM each primer, 4 µL of 5× BDT‐seq buffer, 1.2 µL of BDT 3.1, and 8.6 µL of molecular grade water. The PCR tubes were then placed into a thermocycler under the following conditions: initial denaturation at 95°C for 1 min, annealing again for 30 s at 96°C, annealing at 50°C for 15 s for 35 cycles, and a final extension for 4 min at 60°C, after which the samples were held at 4°C.

Following the cycle sequencing, the sequences were purified using the ethanol/EDTA/sodium acetate precipitation method. The following were directly added to 20 µL of the sample: 10 µL of 125 mM EDTA, 10 µL of 3 M sodium acetate, and 300 µL of absolute ethanol in a 1.5 mL volume of Eppendorf tube. The mixture was then vortexed and maintained at room temperature for 15 min. The samples were centrifuged at 15,000 rpm for 10 min at 4°C. The supernatant was discarded, and 450 µL of 70% ethanol was added, mixed gently, and centrifuged in a similar fashion. Finally, the supernatant was completely removed, and the analyte was dried at room temperature for approximately 10 min and stored at −20°C. Then, the DNA was resuspended in 20 µL of formamide solution and sequenced using an automated ABI 3500xL Genetic Analyzer (Applied Biosystems) according to the manufacturer's instructions.

### 2.8 HIV‐1 Sequence Analysis

The ABI PRISM 3500 Genetic Analyzer (Applied Biosystems) generated sequences of the HIV‐1 pol gene, which were collected and transferred to another computer for further analysis. The exported sequences were edited using Geneious Prime (version 2023.1.1) software (https://www.geneious.com/academic). During editing, the ends of poor‐quality sequences were trimmed at an error probability limit of 0.05, and then the reads were assembled using “the de novo assembly” method to produce one contig sequence. This step was followed by the manual editing of the assembled reads/contigs for ambiguities, gaps, deletions, and insertions of nucleotides to generate consensus sequences exported in FASTA format, making them ready for further sequence analysis.

### 2.9 Genotypic Drug Resistance and Phylogenetic Analysis

Mutations in the HIV‐1 pol gene (protease (PR) and reverse transcriptase (RT)) associated with resistance to PR and RT inhibitors were determined using the Stanford HIV‐1 drug resistance database (version 9.4, https://hivdb.stanford.edu/hivdb), the Calibrated Population Resistance (CPR) tool, and the 2022 updated International Antiviral Society (IAS‐USA) mutation list (http://www.iasusa.org) [27] to investigate HIV‐1 PDR. Sequence quality was checked using an online sequence quality control tool found in the Los Alamos HIV sequence database (https://www.hiv.lanl.gov/content/sequence/QC/index.html). The DRMs were classified as conferring high, intermediate, low, and potential low‐level resistance using the algorithm described in the Stanford HIV‐1 DR database and the IAS‐United States report. In this study, however, only sequences classified as having low‐, intermediate‐, or high‐level resistance were considered for PDR prevalence estimates.

The reference sequences of HIV‐1 Group M (subtypes A‒K) were retrieved from the Los Alamos HIV sequence database (http://www.hiv.lanl.gov) and aligned with the generated sequences to confirm the HIV‐1 subtype. Moreover, the HIV‐1 subtype was determined using online HIV‐1 subtyping tools, including REGA version 3.0 from the Stanford HIVdb (https://hivdb.stanford.edu/hivdb) and the recombinant identification program (RIP). Phylogenetic inference was performed by the maximum‐likelihood method and the general time reversible (GTR) model using PhyML version 3.0 [28]. One thousand bootstrap replicates of multiple alignments using the ClustalW algorithm were used to assess the phylogenetic robustness of the tree [28]. The best substitution model, GTR+G+I, was chosen based on the BIC and AIC. Visualization of the phylogenetic tree was performed with the interactive tree of life (iTOL) version 6.5 software (https://itol.embl.de/tree/). The nucleotide sequences were submitted and deposited in the international GenBank database under accession numbers OR364623–OR364696.

### 2.10 Data Analysis

Data entry was performed using EpiData Manager, version 4.6.0.4, and then the data were exported to STATA/SE version 14.0 for analysis (StataCorp LP, College Station, USA). The baseline characteristics of the study participants were summarized using descriptive statistics. Bivariable and multiple logistic regression analyses were used to identify baseline factors associated with pretreatment drug resistance and the molecular transmission network at ART inception. All variables with crude odds ratios (CORs) of *p* ≤ 0.25 were pooled into adjusted odds ratios (AORs). In multiple logistic regression, a variable with a *p* value < 0.05 was considered a statistically significant factor with outcomes at the 95% confidence level.

## 3 Results

In this study, ART‐naive individuals newly diagnosed with HIV and their baseline viral load > 1000 copies/mL were eligible for HIV‐1 pol region sequence. The genotyping success rate was 78.4% (69/88). Among the 69 patients whose samples were successfully genotyped and included in this analysis, 61% were female, and the median age at enrollment was 35 (interquartile range (IQR): 28–45) years. Details of the participants' characteristics have been published elsewhere [29, 30].

### 3.1 Prevalence and Patterns of Drug Resistance

Overall, 24.6% (17/69) of the sequences had at least one or more drug resistance‐associated mutations. However, according the CPR tool 14.5% (10/69) of the sequences with one or more surveillance drug resistance mutations (SDRMs). According to the WHO drug resistance level criteria and the IAS DRM list, the overall prevalence of PDR among newly diagnosed individuals was 21.7% (15/69) (95% CI: 12.7%–33.3%) (Table 1). Table 1 summarizes the prevalence of PDR for existing ARTs with their baseline viral load.

**Table 1:** PDR mutations and level of resistance to common ARVs at ART initiation according to either the Stanford University HIV‐DR database algorithm or CPR tool among newly diagnosed PWH in eastern Ethiopia, 2020/2021.

Resistance category	Sample ID	DRMs	Age/sex	Viral load	Resistance level	Resistance to ARV drugs
**PIs**						
	OR364641	L90M	50/F	48,044	L	ATV/r, LPV/r
	OR364645	F53L	28/M	252,983	PL*	ATV/r
	OR364664	**M46L**	47/F	412,840	PL*	ATV/r, LPV/r
	OR364687	**I54S**	45/F	7780	L	ATV/r, LPV/r
**NRTIs**						
	OR364642	D67G	45/F	4,102,070	L	AZT
	OR364685	**D67G**	23/M	67,956	L	AZT
	OR364648	**L210W**	30/F	431,781	L	AZT
	OR364649	K65R	46/M	1,604,560	I	ABC, FTC, 3TC, TDF
**NNRTIs**						
	OR364637	**K103N**	35/F	80,636	H	EFV, NVP
	OR364678	**K103N**	40/F	147,700	H	EFV, NVP
	OR364653	**G190A**	35/M	345,136	L	RPV
					I	EFV
					H	NVP
	OR364677	**G190A**	36/F	43,565	L	RPV
					I	EFV
					H	NVP
	OR364666	E138A	34/M	1,955,921	L	RPV
	OR364674	E138A	35/F	4651	L	RPV
	OR364681	E138A	30/F	21,194	L	RPV
	OR364683	**K101E**	43/M	61,563	L	DOR, EFV, ETR
					I	NVP, RPV
**NRTIs + NNRTIs**						
	OR364647	**L210W**	22/F	148,746	L	AZT
		V108I, **Y188C**			L	DOR
		V108I			I	DOR
		V108I, **Y188C**			H	EFV, NVP

*Note:* The mutations in bold are SDRMs only considered by the CPR tool.

Abbreviations: 3TC, lamivudine; ABC, abacavir; ATV/r, atazanavir/ritonavir; DOR, doravirine; EFV, efavirenz; ETR, etravirine; F, female; FTC, emtricitabine; H, high‐level resistance; I, intermediate resistance; L, low‐level resistance; LPV/r, lopinavir/ritonavir; M, male; NNRTIs, nonnucleoside reverse transcriptase inhibitors; NRTI, nucleoside reverse transcriptase inhibitors; NVP, nevirapine; PIs, protease inhibitors; PL*, potential to low‐level resistance and not included to determine PDR prevalence; RPV, rilpivirine; SDRMs, surveillance drug resistance mutations.

The highest prevalence of PDR was observed for NNRTIs (13.04%, 9/69) and 60% (9/15) among the individuals with PDR mutations. NRTIs and PIs DRMs were detected in 7.2% (5/69) and 2.9% (2/69) of the patients, respectively. Both NRTIs and NNRTIs jointly accounted for one‐fourth of the PDR (18.8%, 13/69) and 86.7% (13/15) of the individuals with PDR mutations. Mutations at codons 188 [Y188C] and 210 [L210W] resulted in mutual drug resistance to NNRTIs and NRTIs in one individual (1.4%). Two PI‐associated mutations [L90M and I54S] conferring low‐level resistance to ATV/r and LPV/r were detected in one participant each, accounting for 1.4% of the total. In addition, two mutations (M46L and F53L) conferring potential low‐level resistance to PI‐based ARVs were found once in the study participants.

**Figure 1:**
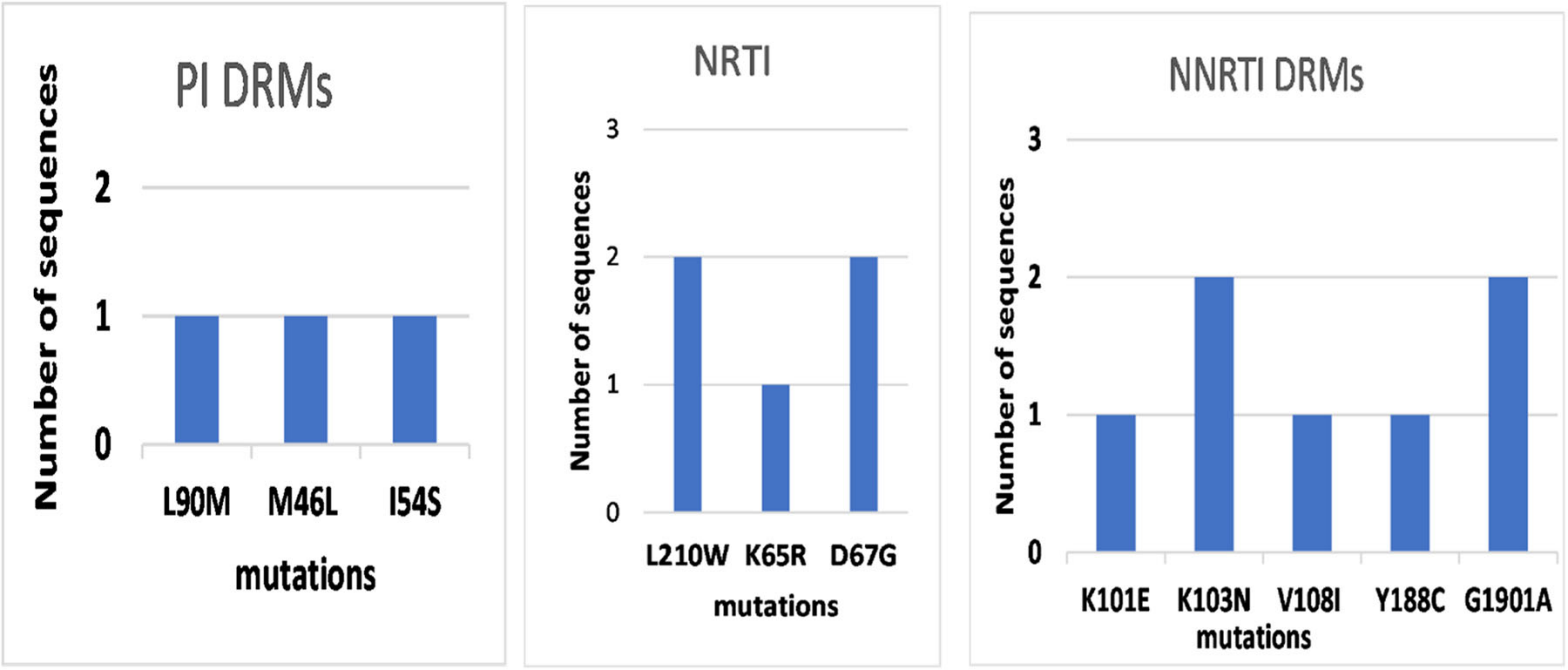
Major DRMs associated with drug resistance in the HIV‐1 pol gene.

Among the participants with PDR, the prevalence of mutations conferring intermediate or high‐level resistance to NNRTIs and NRTIs was seen in 10.1% (7/69). The NNRTI mutation; E138A (4.3%), was observed in the three cases and it is polymorphic mutation in HIV‐1 subtype C but not classified as an SDRM. It is associated with decreased susceptibility to RPV. Both K103N and G190A were found in two (2.8%) of the SDRM patients. The thymidine analog mutations (TAMs) D67G and L210W were detected in two (2.8%) sequences (Figure 1 and Table 1).

**Figure 2:**
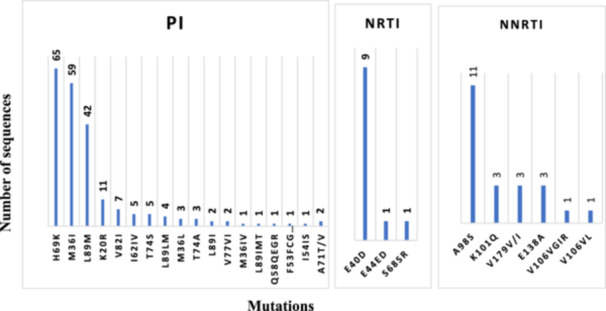
Minor DRMs or polymorphisms associated with the HIV‐1 pol gene.

The most prevalent minor drug‐related PI accessory mutations found were at positions H69K (94.2%), M36I (85.5%), and L89M (60.8%). Minor mutations or polymorphisms such as E40D and A98S were commonly observed in the RT sequences (Figure 2).

### 3.2 Factors Associated With Pretreatment HIV‐1 Drug Resistance

Table 2 shows an analysis of the individuals' baseline characteristics and their HIV‐1 genotypic drug resistance. At baseline, there was a greater proportion of PDR among participants aged above 30 years (80.0%), female (73.3%), and bedridden (40.0%). Approximately 47% of the individuals who had PDR mutations at ART initiation had a viral load greater than 100,000 copies/mL. Multivariable logistic regression analysis revealed that the only baseline factor significantly associated with PDR was a bedridden functional status (AOR: 5.4; 95% CI: 1.53–30.7).

**Table 2:** PDR and baseline characteristics among individuals newly diagnosed with HIV‐1 in eastern Ethiopia, 2020/2021.

Characteristics		PDR					
	*N* (%)	Yes, *n* (%)	No, *n* (%)	COR (95%CI)	*p* value	AOR (95% CI)	*p* value
Age groups (in years)							
≤ 29	21 (30.4)	2 (13.3)	19 (35.2)	1.00		1.00	
> 30–44	28 (40.6)	9 (60.0)	19 (35.2)	4.5 (0.85–23.64)	0.076	4.7 (0.39–55.6)	0.221
≥ 45	20 (28.9)	4 (26.7)	16 (29.6)	2.37 (0.38–14.7)	0.352	2.3 (0.15–31.8)	0.547
Sex							
Male	27 (39.1)	4 (26.7)	23 (42.6)	1.00		1.00	
Female	42 (60.9)	11 (73.3)	31 (57.4)	2.04 (0.57– 7.2)	0.250	2.5 (0.34–18.1)	0.370
Marital status							
Unmarried	11 (15.9)	1 (6.7)	10 (18.5)	1.00		1.00	
Married	28 (40.6)	5 (33.3)	23 (42.6)	2.17 (0.2– 21.1)	0.503	1.2 (0.06–22.3)	0.882
Separated/divorced	18 (26.1)	6 (40.0)	12 (22.2)	5.0 (0.51–48.7)	0.166	9.1 (0.37–224.0)	0.174
Widowed	12 (17.4)	3 (20.0)	9 (16.7)	3.33 (0.29–38.1)	0.333	1.7 (0.06–48.8)	0.744
Occupational status							
Government employee	13 (18.8)	2 (13.3)	11 (20.4)	1.00		1.00	
Farmer and housewife	10 (14.5)	4 (26.7)	6 (11.1)	3.67 (0.51–26.2)	0.196	6.1 (0.39–93.2)	0.194
Merchant	17 (24.6)	2 (13.3)	15 (27.9)	0.73 (0.08–6.04)	0.773	1.28 (0.09–16.4)	0.849
Daily laborer and jobless	20 (28.9)	4 (26.7)	16 (29.6)	1.37 (0.21–8.85)	0.738	1.52 (0.16–14.4)	0.715
Other	9 (13.0)	3 (20.0)	6 (11.1)	2.75 (0.35–21.3)	0.333	7.03 (0.4–110.7)	0.165
Functional status							
Working	40 (57.9)	6 (40.0)	34 (62.9)	1.00		1.00	
Ambulatory	16 (23.2)	3 (20.0)	13 (24.1)	1.31 (0.28–6.01)	0.730	4.49 (0.5–40.2)	0.179
Bedridden	13 (18.8)	6 (40.0)	7 (12.9)	4.85 (1.2–19.57)	**0.026**	5.4 (1.53–30.7)	**0.018**
WHO clinical stage							
Stage I and II	29 (42.0)	6 (40.0)	23 (42.6)	1.00			
Stage III and IV	40 (57.9)	9 (60.0)	31 (57.4)	1.1 (0.34– 3.56)	0.857		
Baseline VL (copies/mL)							
≤ 100,000	33 (47.8)	8 (53.3)	25 (46.3)	1.00			
100,001–500,000	25 (36.2)	4 (26.7)	21 (38.9)	1.41 (0.15–2.25)	0.446		
> 500,000	11 (15.9)	3 (20.0)	8 (14.8)	1.17 (0.24–5.50)	0.841		

*Note:* Bold values indicate significant association.

### 3.3 Circulating HIV‐1 Subtypes and Phylogenetic Analysis

**Figure 3:**
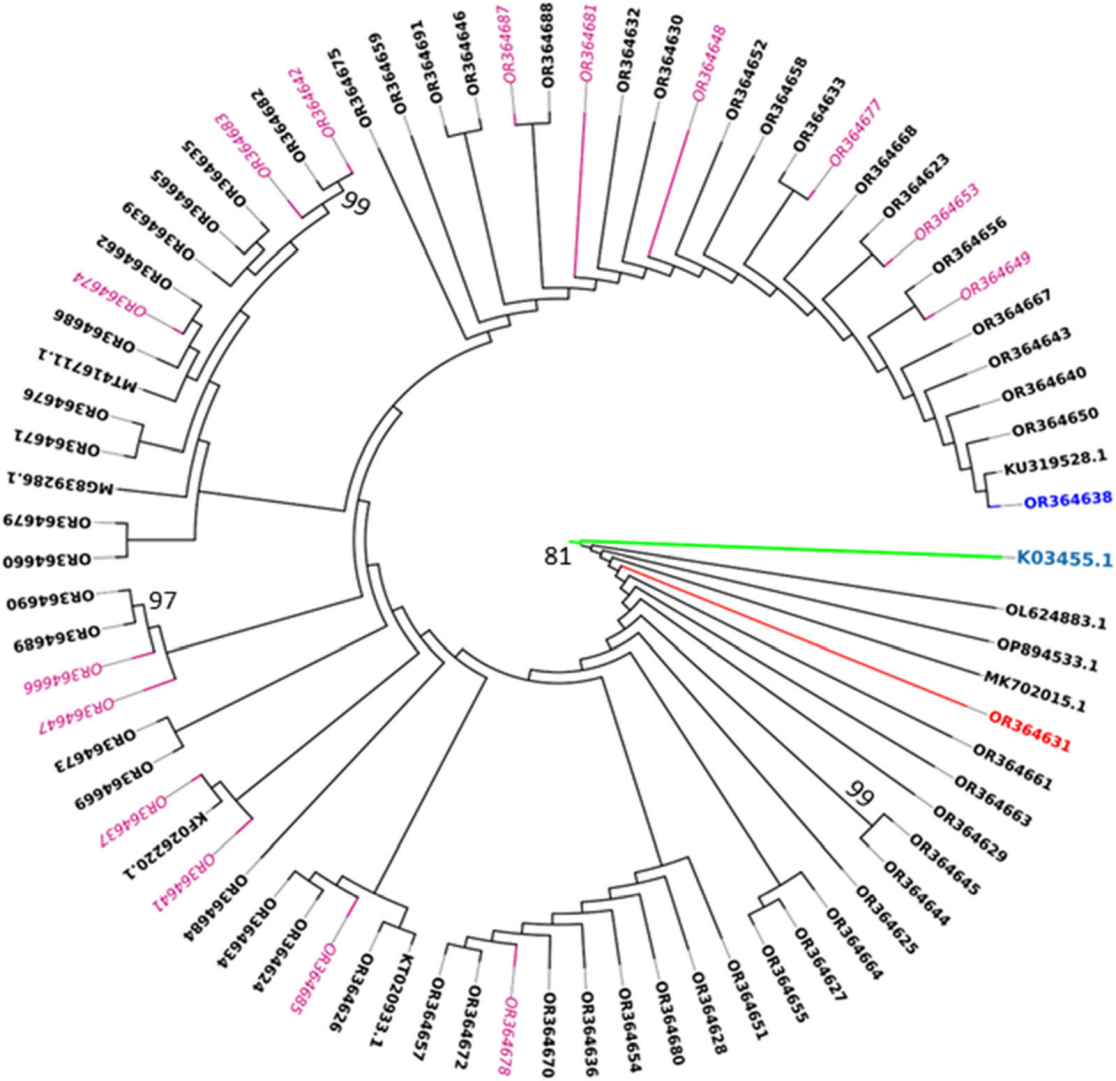
Maximum likelihood phylogenetic tree of the HIV‐1 Pol region using the PhyML method and the GTR nucleotide substitution model.

Subtyping revealed that the most common circulating HIV‐1 subtype was subtype C (97.1%). Two isolates were recombinants of A1C (A1(21.7%), C (78.3%) and CF1 (C (60.6%), F1(39.4%)) accounting for 1.4% of the samples in each case. All sequences were closely related and formed monophyletic transmission clusters with a bootstrap value of 81%. With a bootstrap value ≥ 95%, three clusters (8.7%, 6/69) of closely related transmissions were found (see Figure 3). A phylogenetic relationship with subtype C isolated from different parts of Ethiopia, where subtype C circulates, was also observed.

This analysis involved 69 nucleotide sequences (PR and RT nucleotide sequences) along with reference sequences from the Los Alamos HIV database. Those with GenBank accession numbers OR364623–OR364696 are sequences from this study, and sequences with PDR mutations are highlighted with a pink node and italicized, while one (Subtype A1C) is highlighted with a red node, and CF1 is highlighted with blue. The remaining HIV‐1 reference sequences were retrieved from the Los Alamos HIV database. The following reference sequences were used: KU319528.1 (subtype C‐Ethiopia), MG839286.1 (subtype C‐Ethiopia), MT416711.1 (subtype C‐Ethiopia), KF026220.1 (subtype C‐Ethiopia), and KT020933.1 (subtype C‐Ethiopia). The remaining sequences—K03455.1 (HXB2‐France), MK702015.1 (Camerron), OP894533.1 (Ghana), and OL624883.1 (Brazil)—are non‐C subtypes. Only bootstrap values > 70% are indicated at each node.

## 4 Discussion

This is the first report from eastern Ethiopia to identify pretreatment HIV‐1 DR mutations and the circulating HIV‐1 subtypes among the community. This study revealed a high level of PDR mutations, mainly to NNRTIs. The dominant circulating HIV subtype was clade C.

This study reported high prevalence (21.7%) of PDR as compared to reports from different parts of Ethiopia [20, 23]. However, the current findings were comparable to those of one study conducted in Ethiopia (when only DRMs detected by the CPR tool considered) (14%) [21] and other parts of the world, such as Cameroon (15%) [31], the Republic of Guinea (25%) [32], and Shenzhen, China (23.1%) [33]. Our result was lower than that of a study in Sierra Leone, which reported a PDR of 36.7% in the ART‐naive group [34].

The possible explanation for the increase in PDR in the current study could be attributed to the accumulation of DRMs spread locally by individuals failing ART or with transmitted DRMs. Additionally, prolonged epidemics and access to ART may contribute to increased PDR over time, as seen in pooled analyses of PDR trends in Ethiopia [25] and Mozambique [35]. Another possible explanation is that, unlike the majority of previous studies conducted in Ethiopia, the current study covered wide regions with different cultural and sociodemographic backgrounds across eastern Ethiopia, reflecting the burden of the PDR in the area. Moreover, due to the paucity of previous data from the area, the direction of the PDR trends in eastern Ethiopia is unknown.

In this study, the most common PDR mutations were associated with NNRTIs, followed by NRTIs and PIs. This finding corroborates those of several studies conducted in Ethiopia and elsewhere. This study revealed a greater prevalence of NNRTI‐associated mutations (13%) than did previous PDR studies done among ART‐naive individuals with HIV in Ethiopia [20]. Among the identified NNRTI‐associated mutations, the most common were E138A, K103N, and G190A, followed by K101E, V108I, and Y188C. This finding is in agreement with previous reports from Ethiopia [19, 20].

In this study, D67G and L210W mutations selected by the thymidine analog zidovudine (AZT) were observed equally in the two samples. In addition, in one patient, the K65R mutation resulted in intermediate‐level resistance to abacavir (ABC), emtricitabine (FTC), lamivudine (3TC), and TDF. Compared with this finding, higher proportions of these mutations were reported in a previous study conducted among female sex workers in Ethiopia [22]. Thus, appropriate measures should be taken to address PDR because these drugs are commonly used as the backbone of treatment regimens in resource‐limited settings, including Ethiopia, even during the DTG era [36].

According to the Stanford University HIVDR algorithms, the protease inhibitor mutations observed in this study were L90M, I54S, M46L, and F53L. The observed frequency was relatively greater, and the mutation patterns were also different from those in a previous study [20]. However, the M46L mutation has also been reported in one individual in Ethiopia who failed first‐line ART but was naive to protease inhibitors [37]. Similarly, a study in Mozambique revealed that M46L and L90M PI‐related mutations that are nonpolymorphic are associated with reduced susceptibility to atazanavir (ATV) and lopinavir (LPV) [35, 38].

According to the final model of the analysis, the only baseline factor independently associated with the PDR was the functional status (bedridden with AOR = 5.4) of the individuals with HIV at treatment initiation. This finding was in line with a recent report from another region of Ethiopia [39]. This might be related to the low immune status of the patients due to opportunistic infections or comorbidities that increase the likelihood of viral replication in the body. A low CD4+ cell count was significantly associated with PDR mutations in a previous study [22]. Although not significant in this study, high VL before ART initiation has been reported to be associated with DRMs [40]. In our study, regardless of the DRM status, the median baseline HIV RNA among those bedridden was greater than that among those with both working and ambulatory functional status (152,899 vs. 25,194.5 and 50,723.5 copies/mL, respectively). The mean VL of the bedridden study participants was significantly greater than that of the working participants (547,733.3 vs. 112,817.6, F test: 7.86, *p* = 0.004). Consequently, this high viremia for longer periods may lead to the accumulation of resistance‐associated mutations, decreased CD4+ T‐cell counts and increased viral load [40, 41].

Genetic diversity analysis of the pol region sequences indicated that the HIV‐1 subtype C virus was the predominant clade in eastern Ethiopia. Several studies from Ethiopia consistently described the Ethiopian HIV epidemic as being predominated by HIV‐1 subtype C (97–100%) [23, 39]. This finding is consistent with the literature on HIV subtypes circulating in other Eastern and Southern African countries, such as Mozambique [35], Malawi [42], and Botswana [43]. Subtype HIV‐1 C accounts near to 50% of HIV global infection [13]. With this rate of global expansion of HIV‐1 subtype C, controlling and monitoring of HIV‐1 drug resistant variants are critical. Particularly, in LMICs where there is high prevalence of HIV, limited resources, lack of routine genotypic resistance, and multifactorial factors contribute for the drug resistant strains [44]; tailored interventions and appropriate measures are needed to overcome the problem.

Despite low in number, recombinant A1C and CF1 were observed. Recombinant A1C (0.3%) was also reported in Ethiopia [39] and Kenya (2.7%) [45]. Moreover, Eastern Africa is mostly affected by HIV‐1 subtypes D, C, A, and by a high proportion of URFs [13]. Thus, this may also contribute to the circulating HIV‐1 recombinant variants. However, to the best of our knowledge, no previous Ethiopian studies have reported recombinant CF1. This recombinant has a low prevalence and has been reported in different parts of Brazil [46, 47]. The occurrence of these other variants indicates the possible introduction of other HIV‐1 subtypes from other neighboring countries or non‐neighboring countries due to globalization effects. Specifically, eastern Ethiopia is the main corridor of transportation for internationally traded goods from neighboring countries and ports of Djibouti, involving HIV‐vulnerable groups of people categorized as key and priority populations in national guidelines, such as long‐distance truck drivers [48]. In addition, high proportions of in‐and‐out migrants from countries such as Djibouti are common in eastern Ethiopia based on seasonal variation. These factors directly or indirectly increase the likelihood of HIV‐1 genetic diversity and the introduction of new variants of HIV‐1 circulating in Ethiopia; thus, further ongoing investigations are required to develop preventive measures for the influx of new subtypes.

The strength of this study is that the PDR was determined in eastern Ethiopia, a large area where there are no previous data. This study also has certain drawbacks. The sample size was relatively small. The Sanger dideoxy sequencing method used for genotyping HIV strains may not detect a resistant minority variant if it is present in less than 20% of the total viral population [49]. This may have resulted in underestimation of the PDR.

## 5 Conclusions

This study examined the PDR mutations and circulating HIV‐1 subtypes among individuals newly diagnosed with HIV in eastern Ethiopia. High levels of pretreatment HIV‐1 drug resistance, predominantly to NNRTIs, are found. Moreover, resistance to NRTIs and PIs is moderate and low, respectively, with little increase. The bedridden status of new patients at the time of ART initiation is significantly associated with PDR and thus requires special attention during clinical follow‐up. Thus, periodic HIV‐1 drug resistance studies are crucial for monitoring and evaluating trends in PDR and assessing context‐specific factors related to baseline DRMs. Moreover, the observed PDR to NRTIs and PIs warrants further surveillance to evaluate the long‐term implications of pre‐existing resistance to the backbone of the dolutegravir‐based regimen, as well as the effect of a prolonged accumulation of resistance on the current ART regimens and HIV control strategies.

## Author Contributions

**Abdella Gemechu:** conceptualization, data curation, formal analysis, funding acquisition, investigation, methodology, project administration, resources, software, supervision, validation, visualization, writing – original draft, writing – review and editing. **Adane Mihret:** data curation, supervision, writing – review and editing. **Mesfin Mengesha:** data curation, writing – review and editing. **Dawit Hailu Alemayehu:** data curation, writing – review and editing. **Eleni Kidane:** data curation, writing – review and editing. **Abraham Aseffa:** data curation, supervision, writing – review and editing. **Rawleigh Howe:** data curation, writing – review and editing. **Berhanu Seyoum:** data curation, supervision, writing – review and editing. **Andargachew Mulu:** data curation, formal analysis, funding acquisition, methodology, project administration, software, supervision, writing – review and editing.

## Ethics Statement

Ethical approval was obtained from the Institutional Health Research and Ethics Review Committee of the College of Health and Medical Sciences, Haramaya University (IHRERC: Ref. No. IHRERC/202/2020) and the AHRI/ALERT Ethics Research Committee (AAERC: PO/22/20) before the commencement of data collection.

## Consent

Informed voluntary signed consent was obtained from each study participants. Confidentiality of the study participants were assured and the information would be kept private.

## Conflicts of Interest

The authors declare no conflicts of interest.

## Transparency Statement

The lead author Abdella Gemechu affirms that this manuscript is an honest, accurate, and transparent account of the study being reported; that no important aspects of the study have been omitted; and that any discrepancies from the study as planned (and, if relevant, registered) have been explained.

## Supporting information


**Table S1. List of in‐house primers used for HIV‐1 pol gene PCR ampliﬁcation, cDNA synthesis, and thermal cycling**.

## Data Availability

The data that support the findings of this study are available on request from the corresponding author. In addition, Supporting file and sequence data can be accessed from the database (GenBank database under accession numbers OR364623–OR364696). The data are not publicly available due to privacy or ethical restrictions.
