# Supplementary material for: Pretreatment HIV‐1 Drug Resistance Among Newly Diagnosed People in Eastern Ethiopia
Source: Health Sci Rep. 2025 Apr 16;8(4):e70672. doi: 10.1002/hsr2.70672 (PMC12000924; doi:10.1002/hsr2.70672)
Supplement: Supplementary file 1 — Table S1. List of in‐house primers used for HIV‐1 pol gene PCR ampliﬁcation, cDNA synthesis, and thermal cycling. [file HSR2-8-e70672-s001.docx]

**Table S1. List of in-house primers used for HIV-1 pol gene PCR ampliﬁcation, cDNA synthesis, and thermal cycling.**

| Parameter |  | cDNA synthesis | | First round PCR | | Nested PCR | |
| --- | --- | --- | --- | --- | --- | --- | --- |
|  | **Category** | **Location on HXB2** | **Sequence (5’→3’)** | **Location on HXB2** | **Sequence (5’→3’)** | **Location on HXB2** | **Sequence (5’→3’)** |
| Primer/s | Forward |  |  | 1826-1849 | TGATGACAGCATGTCAGGGAGTGG | 2150-2167 | AGCCAACAGCCCCACCAG |
|  | Reverse | 3630-3649 | TGTTTTACATCATTA GTG TG | 3555-3583 | GGCTCTTGATAAATTTGATATGTCCATTG | 3514-3539 | CTGTATTTCTGCTATTAAGTCTTTTG |
| Master mix (MM) Components | | **Components 1:**  HIVRevINT primer=2 µL  25 mM DNTP=1 µL  MGW=2 µL  65°C for 5 min, 1 min on ice. Then,  **Components 2:**  5X Superscript IV buffer= 4 µL  100 mM of DTT= 1 µL  RNase/Ribolock= 1 µL  Superscript IV RT= 1 µL  55°C for 10 min & 80°C for 10 min | |  | MGW = 33. µL  10x Buffer (-MgCl2) = 5 µL  50 mM MgCl2= 2.7 µL  10 mM dNTP’s= 1 µL  10 µM HIVPCRFor Primer= 1 µL  10 µM HIVPCRRev Primer= 1 µL  Taq Polymerase= 0.2 µL  **45 µL of MM + 5 µL Template DNA** |  | MGW= 34. µL  10x Buffer (-MgCl2) = 5 µL  50 mM MgCl2= 2.7 µL  10 mM dNTP’s= 1 µL  10 µM HIVseqA Primer= 1 µL  10 µM HIVseqH Primer= 1 µL  Taq Polymerase= 0.2 µL  **47 µL of MM + 3 µL Template DNA** |
| PCR thermal cycling conditions | | | |  | 1-Initial Denaturation: 94°C/2 Min; 2-Denaturation: 94°C/30 Sec; 3- Annealing: 54°C/1Min; 4-Elongation: 72°C/1Min; 5-Go to Step 2: 35 X; 6-Final Elongation: 72°C/5 Min; 7- Cooling: 4°C/Forever; 8-End |  | 1-Initial Denaturation: 94°C/2 Min; 2-Denaturation: 94°C/30 Sec; 3- Annealing: 54°C/1Min; 4-Elongation: 72°C/1Min; 5-Go to Step 2: 35 X; 6-Final Elongation: 72°C/5 Min; 7- Cooling: 4°C/Forever; 8-End |

MGW- Molecular grade water (distilled water), RT- reverse transcriptase *All positions are matched to HIV-1 HXB2 (reference sequence) (GenBank Accession number: K03455).
